# Supplementary material for: Association between oral microbiome and seven types of cancers in East Asian population: a two-sample Mendelian randomization analysis
Source: Front Mol Biosci. 2023 Nov 21;10:1327893. doi: 10.3389/fmolb.2023.1327893 (PMC10702768; doi:10.3389/fmolb.2023.1327893)
Supplement: Supplementary file 1 [file DataSheet1.ZIP › Supplementary Materials/MR plots of saliva/saliva═╝/Pancreatic cancer/pheno.435_to_pancreatic cancer_leave_one_out.pdf]

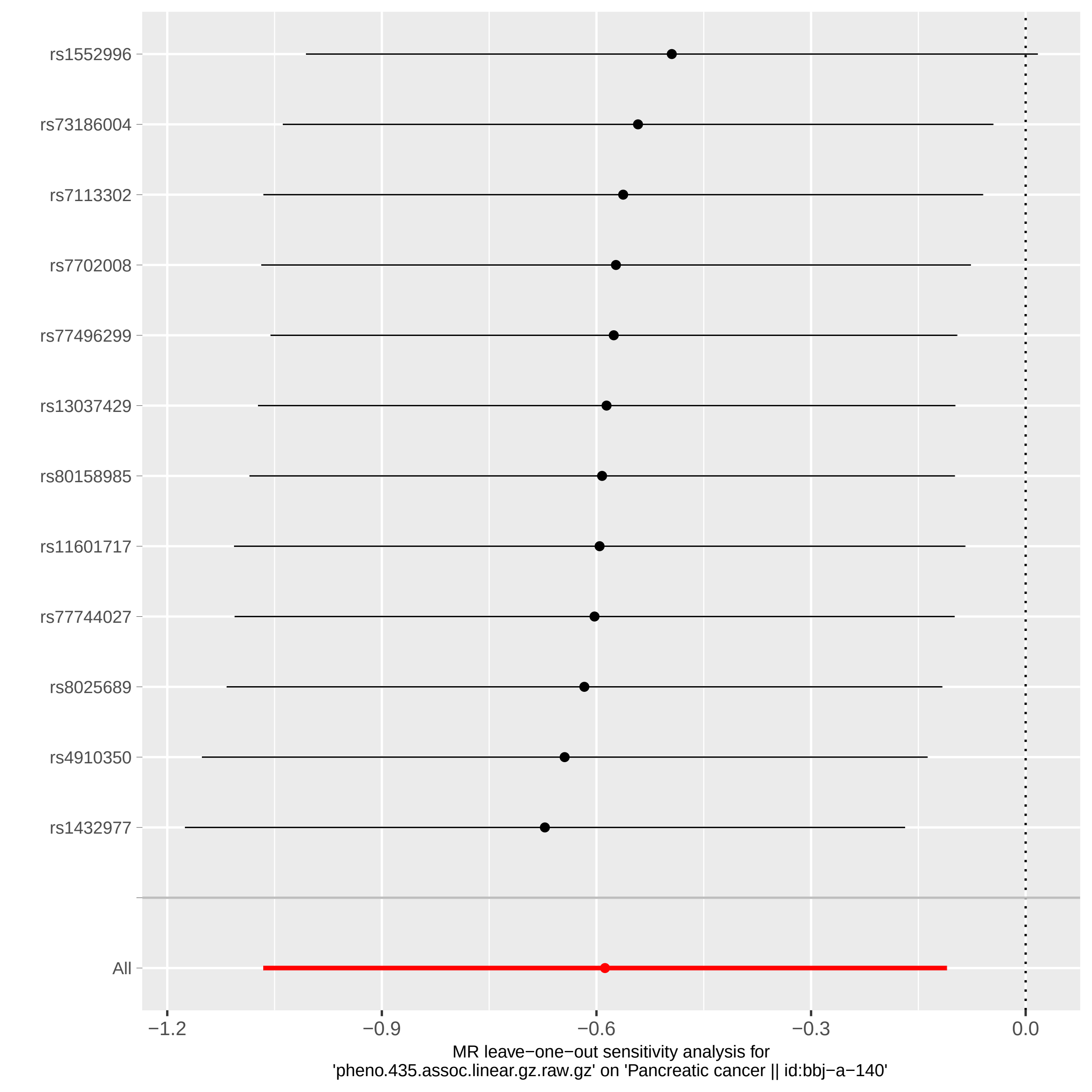
rs1552996
rs73186004
rs7113302
rs7702008
rs77496299
rs13037429
rs80158985
rs11601717
rs77744027
rs8025689
rs4910350
rs1432977
All
−1.2
−0.9
−0.6
−0.3
0.0
MR leave−one−out sensitivity analysis for
'pheno.435.assoc.linear.gz.raw.gz' on 'Pancreatic cancer || id:bbj−a−140'
